# Three-dimensional analysis of the physiological foramen geometry of maxillary and mandibular molars by means of micro-CT

**DOI:** 10.1038/ijos.2017.29

**Published:** 2017-09-08

**Authors:** Thomas Gerhard Wolf, Frank Paqué, Michael Sven Patyna, Brita Willershausen, Benjamín Briseño-Marroquín

**Affiliations:** 1 Department of Operative Dentistry, University Medical Center of the Johannes Gutenberg University Mainz, Mainz, Germany; 2 Department of Endodontology, Division of Preventive Dentistry, Periodontology, and Cariology, Center of Dental Medicine, University of Zürich, Zürich, Switzerland

**Keywords:** apical constriction, final apical file, maxillary and mandibular molars morphology, micro-computed tomography, physiological foramen

## Abstract

The aim of this study was to investigate the physiological foramen diameter, shape and distance between physiological and anatomical apex of maxillary and mandibular first and second molars. Accurate knowledge of the physiological foramina morphology; thus, inherent mechanical shaping technical hindrances, is decisive when taking the corresponding root canal final preparation decision. The morphological dimensions of a total of 1727 physiological foramina were investigated by means of micro-computed tomography. Mean narrow and wide (to a high number, oval) diameters of the physiological foramen were 0.24, 0.22 and 0.33 mm and 0.33, 0.31 and 0.42 mm in mesiobuccal (MB), distobuccal (DB) and palatal (P) roots in maxillary first molars; 0.24, 0.22 and 0.33 mm and 0.41, 0.33 and 0.44 in MB, DB, and P roots in maxillary second molars. Mandibular first molars showed mean narrow and wide diameters of 0.24 and 0.30 mm and of 0.39 and 0.46 mm in mesial (M) and distal (D) roots; second mandibular molars showed 0.25 and 0.31 mm and 0.47 mm in M and D roots. The mean distance between the physiological foramina and anatomical apex was 0.82, 0.81 and 1.02 mm and 0.54, 0.43 and 0.63 mm in MB, DB and P roots of the maxillary first and second molars, respectively. A mean distance of 0.95 mm (M) and 1.05 mm (D) in the first and 0.78 mm (M) and 0.81 mm (D) in the second mandibular molars was observed. Based on the results obtained, assumable recommendations for final preparation size of the physiological foramen were calculated. However, when taking into consideration, the resulting standard deviations of marginal errors must be cautiously considered when taking a final decision in clinical endodontic treatment.

## Introduction

Successful endodontic non-surgical and surgical endodontic treatment requires detailed knowledge of tooth anatomy and morphology.^1–2^ Morphological knowledge of the apical region should be accurate, as instrumentation and filling of root canals is based, to a great extent, on that information. Understanding of the apical area and the tooth root canal morphology is a complex and important condition that the clinical operator needs for making decisions during endodontic treatment.^3^

Root canals should be prepared up to the physiological foramen, also termed apical constriction.^4^ Because of the results of experimental studies and biological principles, instrumentation and obturation beyond the apical foramen should be avoided.^5^ While comparisons of techniques and instruments concerning root canal obturation^6^ have been discussed in the literature, different instrumentation challenges during treatment and re-treatment can occur, when, for example, a multi-constricted or a parallel, tapered^7^ or even no apical constriction^5^ are given. Different authors^1, 4, 7, 8, 9, 10, 11, 12^ have investigated the physiological foramen and have also shown the anatomic variations of the root canal systems. These investigators have employed different research techniques such as clearing technique, scanning electron microscopy, optical microscopy or micro-computed tomography. Micro-computed tomography with software rendering is known as a high-resolution imaging technique that provides a three-dimensional and detailed imaging of the tooth structures.^13, 14, 15, 16^ The present study provides morphological data of the physiological foramen by means of high-resolution micro-computed tomography. The aim of this investigation was to determine the distance between the physiological and anatomical apex, the diameter and the shape of the physiological foramen, as well as to outline a clinically oriented physiological foramen size recommendation for the roots of maxillary and mandibular first and second molars; thus, pursuing an apical shaping size recommendation for these teeth.

## Materials and methods

### Tooth selection

516 extracted human permanent maxillary and mandibular first and second molars were obtained for reasons unrelated to this study. The investigated teeth were maxillary and mandibular first and second molars.^17^ Selection criteria were complete root development, no restoration, no signs of root fracture or resorption, no radicular or coronal caries and no endodontic treatment. The teeth were cleaned of any attached hard and/or soft tissues and calculus by means of an ultrasonic scaler, placed for 1 h in a 3% hydrogen peroxide ultrasonic bath, and then stored in 70% alcohol according to their type and dental arch position. For further investigation of the internal morphology of the pulp chamber floor and root canal entrances (results not reported in this investigation) endodontic access cavities were prepared with a high-speed handpiece and a diamond round bur (801-014; Komet, Lemgo, Germany). When required, ultrasonic tips (CAVI 2-D and 3-D; VDW, Munich, Germany) were used to remove pulp stones in the pulp chamber area. The pulp chambers were rinsed with 1% sodium hypochlorite (60 s) and dried by means of suction.

### Micro-computed tomography morphological analysis

**Figure 1:**
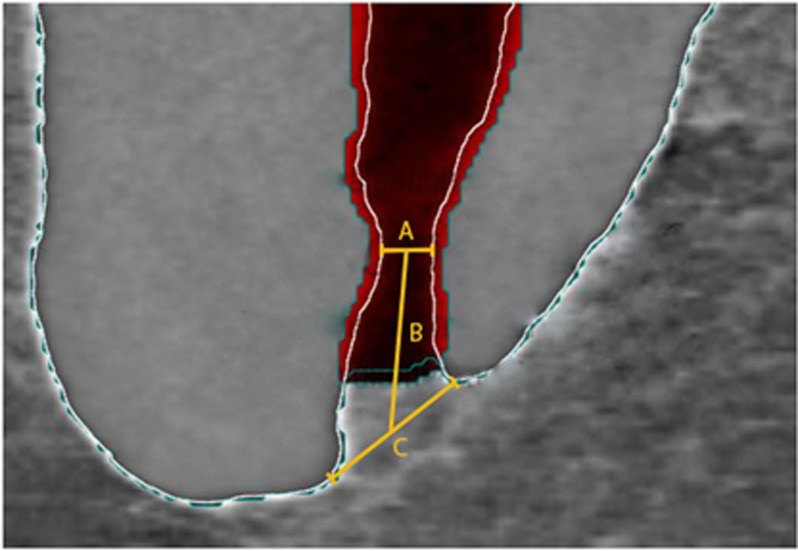
**Micro-computed tomography representation of the distances measured at the apical foramen region**. **A**, diameter (narrow) of the physiological foramen. **B**, distance between the physiological and anatomical foramen. **C**, diameter (wide) of the anatomical foramen.

The teeth were scanned by means of a previously established method^13, 14, 15^ at settings of 70 kV and 114 μA, resulting in 800–1 200 slices per tooth at an isotropic resolution of 20 μm in a desktop micro-computed tomography (μCT) unit (μCT 40; Scanco Medical, Brüttisellen, Switzerland). A specific software (VGStudio Max 2.2; Volumegraphics, Heidelberg, Germany) was used to be able to differentiate the tooth structures. Images were visualized through depiction in dummy colors in 3D reconstructions of the μCT scans obtained. Tooth structures were color coded by means of the rendering software. In this study only the main foramen or foramina was analyzed and was defined as the one which emerged from the main root canal at the apical terminus and in which the measured diameter difference between the multiple foramina, when present, was no less than 0.2 mm.^18, 19, 20^ Furthermore, the distance between the physiological foramen center and anatomical apex, the narrowest and widest diameters as well as the shape of the physiological foramen were determined by means of μCT imaging (Figure 1). The corresponding final apical file size was calculated at two instrument sizes bigger^9^ according to the corresponding measured diameter and its clinical implications discussed. The results are expressed as statistical analysis and according to the sample number; they are expressed through absolute and relative values.

## Results

A total of 1 727 physiological foramina of 516 maxillary and mandibular first and second molars were investigated. The sample distribution is shown in Table 1. The distance between the physiological foramina and anatomical apex, number, diameter and shape of the physiological foramen and determination of the initial apical file; thus, clinical outcome of the physiological diameter, of mandibular and maxillary first and second molars are provided in Tables 2, 3, 4, 5.

## Discussion

**Figure 2:**
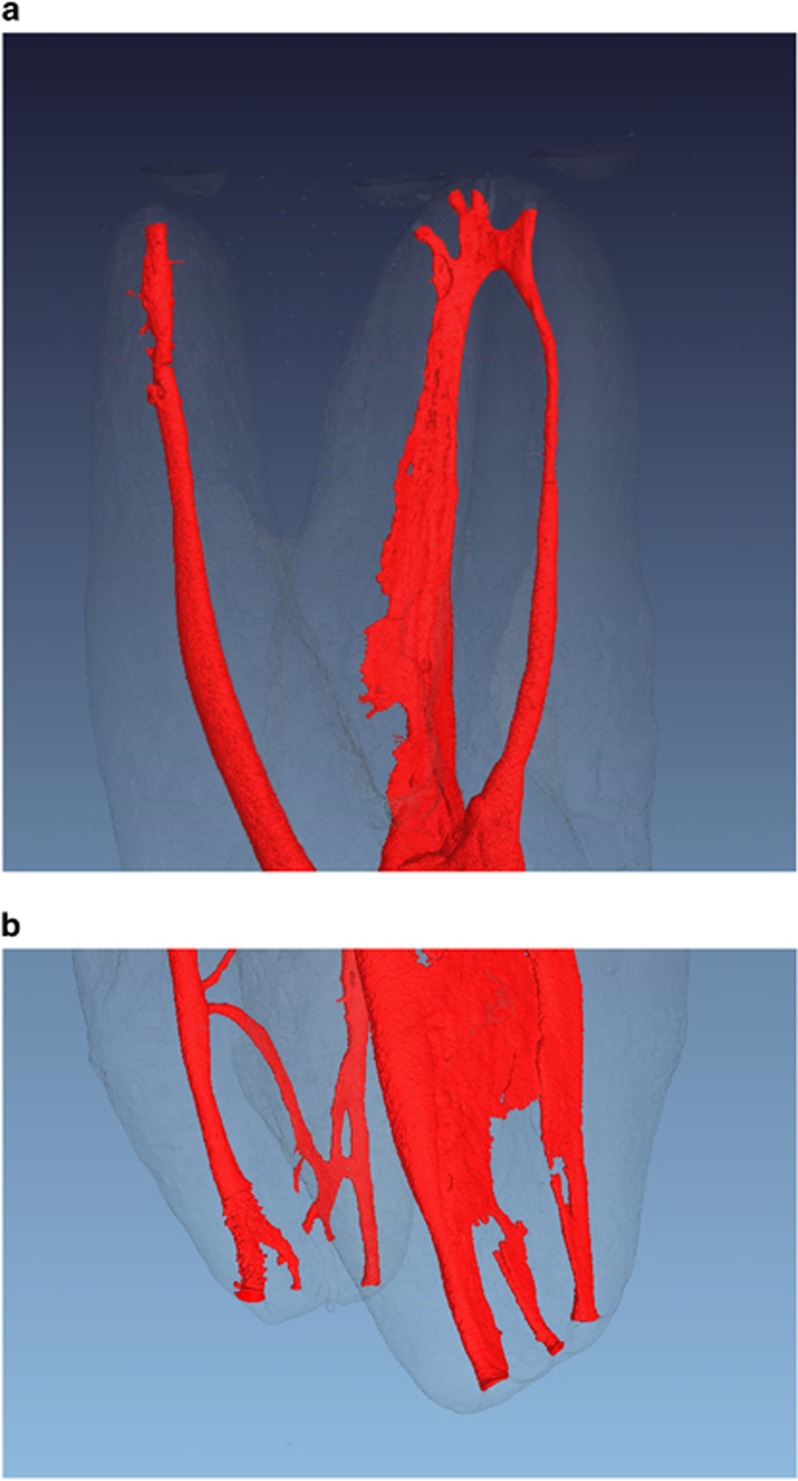
**Micro-computed tomography of a maxillary (a) and mandibular (b) molar, respectively**. It can be denoted the complexity of the root canal system morphology, specially of the apical area where the portals of communication with the periapical tissues clinically demand an accurate preparation an hermitic seal.

Endodontic anatomical knowledge is essential to ensure long-term treatment success; therefore a precise description of the apical area is essential.^1^ Up to the present time, minute data has been lacking concerning the morphology of the terminal part of the root canal system and three-dimensional high resolution techniques prevail (Figure 2a and Figure 2b). For this purpose micro-computed tomography offers a reproducible, non-destructive, non-invasive method for non-clinical *ex-vivo* investigation, providing substantial information about minor structures such as the terminal part of the apical region of teeth, and allowing measurements of the structures examined.^19, 20, 21, 22^ Although scientifically burdensome as evidence, it seems that a large number of researchers would agree that micro-computed tomography provides a greater amount of objective information than conventional two-dimensional optical methods,^10^ scanning microscopy^23^ or the clearing technique.^2^ Therefore, a relatively high number of teeth which had been precisely identified were investigated in the present study by means of micro-computed tomography, allowing a sound statistical evaluation of the sample.

**Figure 3:**
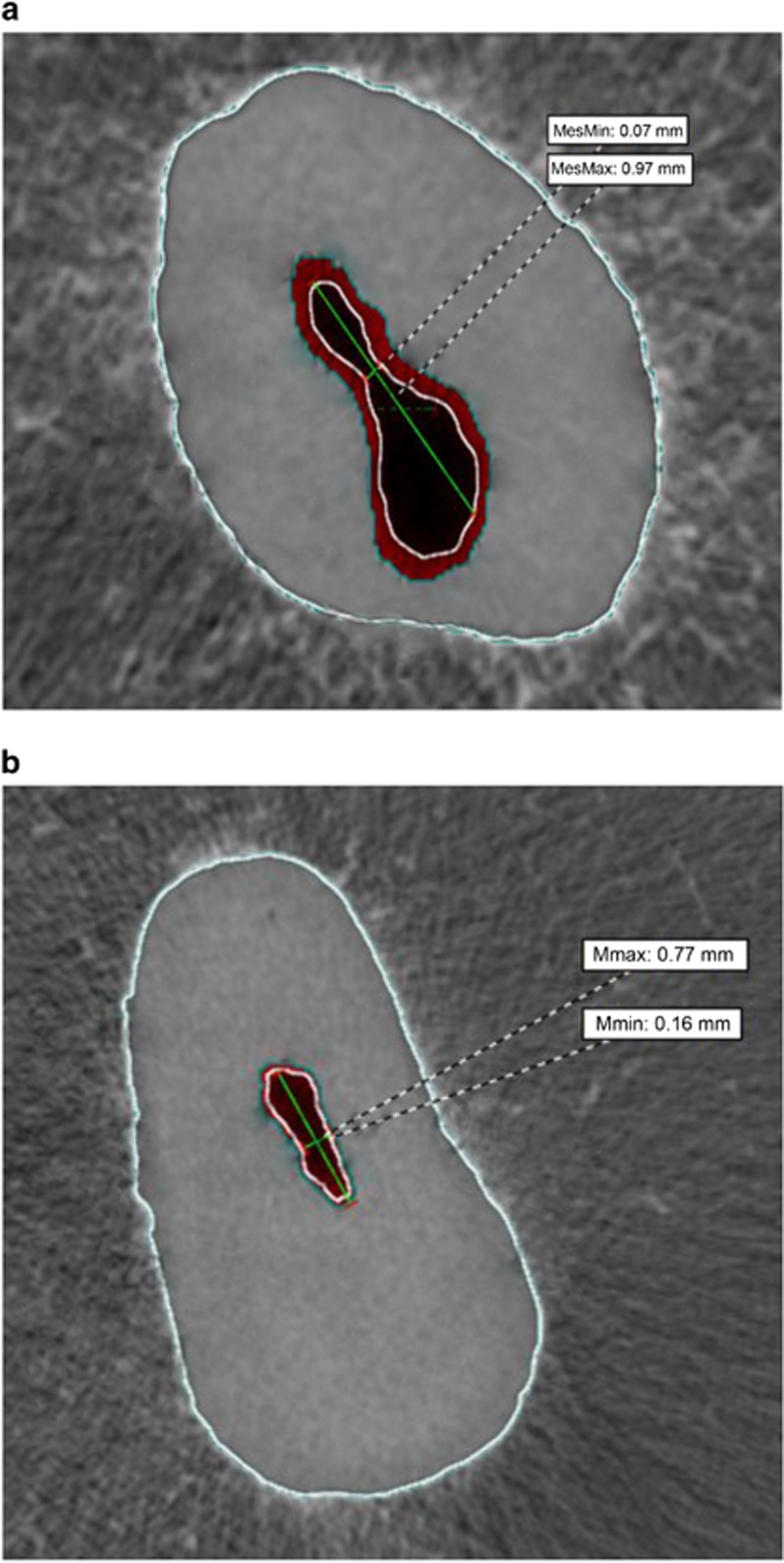
**Micro-computed tomography horizontal plane at the physiological foramen level of the distal and palatal roots of a mandibular (a) and maxillary molar (b), respectively**. In this plane the oval shape of the foramina can be clearly distinguished. The differences between the major and minor diameters of the foramina denote the demanding challenges to achieve a clinically ideal preparation of the foramen. max, maximum; min, minimum.

The physiological foramen, in our opinion wrongly termed “apical constriction” or “cemento-dentinal junction”, has been defined as the narrowest diameter of the root canal.^1^ Actually a cemento-dentin junction can be observed along the entire root and not only in the apical area. Furthermore, an apical constriction cannot be consistently observed in the foramen;^10^ thus, allowing a clear differentiation between the anatomical and physiological foramen. This region has been relatively seldom investigated; however, controversy often surrounds its discussion. Our detailed results of maxillary and mandibular molars are similar to the ones obtained by Kuttler^1^ and Abarca *et al.*^24^ Yet, Kuttler^1^ investigated 82 teeth without mentioning the tooth type and Abarca *et al.*^24^ investigated a total of 174 physiological foramina of maxillary and mandibular first molars. The narrow diameter results by means of a morphometric two dimensional (2D) analysis at the apical level of the distal canals of mandibular second molars reported by Filpo–Perez *et al.*^12^ are higher when compared with ours. These differences could be explained by the different ethnic origins of the teeth investigated or the sample number and the investigation method employed. Our results are also not in agreement with the ones obtained by Simon.^4^ who defined the apical constriction as “critical zone”, and reported that a constriction at the cemento-dentin junction in most roots was not present. In our sample, all roots examined showed a typical or less distinct apical constriction; yet it could always be clearly identified, and thus measured. Nevertheless, we fully agree with different authors^4, 5, 9^ that a significant proportion of success or failure depends on the adequate treatment of this “critical zone” (Figure 3aFigure 3b). Our physiological foramina shape findings are contrary to the ones in maxillary and mandibular incisors, canines and premolars, described by Dummer *et al.*,^7^ such as traditional single constriction, tapering constriction, multi constricted and parallel wall “constriction”. Although in this study, under micro-computed tomography, it was always possible to observe a typical or less clear distinctive physiological foramen (traditional apical constriction) we also agree with this research group that the localization and shaping of the physiological foramen is a clinical demanding task. The differences in results between these two last mentioned investigations and ours could be explained by the different type of teeth investigated, or, in our opinion, mainly by the different research methodologies.

If a final round preparation, as suggested by Weine,^25^ of the physiological foramen is desirable, it would be only possible if the operator were able to perceive its original shape and dimensions. Regardless of the results obtained in this study it would be difficult to assert physiological foramen preparation sizes due to the inconsistent morphology shape involved. If the size of the binding instrument tip (initial apical file) at the physiological foramen is assumed according to the physiological foramen diameter mean values here reported, it is mandatory to point out that such a recommendation, according to the standard deviations obtained, would be wrong from 20% to 58% of the time (Table 5). Furthermore, since the oval shape of the physiological foramen was the most common one found (63% to 81% Table 4), clinically it would be almost impossible with the help of tactile sensitivity alone to determine the size of the wide diameter, which is actually the one that must be determined in order to be able to make the shape of the physiological foramen completely round.

In the literature, the anatomical foramen (apex) has been investigated more often than the physiological foramen (apical constriction). The anatomical foramen, defined in this study as the widest diameter, results (Table 3; Figure 1), showed in the maxillary first molar a 0.33 mm ±0.14 mm diameter in the mesiobuccal (MB), 0.31 mm ±0.12 mm in the distobuccal (DB) and 0.42 mm ±0.14 mm in the palatal (P) root and thus similar to the results reported by Green^26^ concerning maxillary first and second molars together (0.35 mm/mesiobuccal and 0.40 mm/distobuccal and palatal roots), by Abarca *et al.*^24^ concerning maxillary first and second molars as well (0.307 mm/mesiobuccal, 0.320 mm distobuccal and 0.400 mm palatal) and by other researchers^27^ in maxillary teeth (canine 0.353 mm, lateral incisor 0.292 mm and central incisor 0.298 mm). The results reported by Mizutani *et al.*^28^ are slightly higher (0.375 mm/canines, 0.390 mm/lateral incisors and 0.429 mm/central incisors) than ours. Our results of the mandibular first molar (0.37 mm ±0.21 mm/mesial and 0.46 mm ±0.20 mm/distal roots) are lower than those of Green (0.5 mm/mesial and 0.65 mm/distal),^26^ Green^29^ (0.52 mm/mesial and 0.64 mm/distal) and Wu *et al.*,^5^ who measured the median of canal diameters 1 mm “from the apex” (0.40 mm/mesiobuccal, 0.38 mm/mesiolingual and 0.46 mm/distal root). While Morfis *et al.*^23^ reported slightly lower values (0.257 mm/mesial and 0.392 mm/distal root), the results of Abarca *et al.*^24^ are closer to those in the present study (0.311 mm/mesial and 0.360 mm/distal root). These differences could be attributed to the sample type, origin and/or number as well as to the research methodology. To the best of our knowledge there is no data available, concerning the anatomical foramen, on the mandibular second molar in the literature.

The distance between the physiological foramen (apical constriction) and anatomical apex has scarcely been investigated in maxillary^10^ and mandibular molars.^30^ The results of the maxillary first (0.82 mm MB, 0.81 mm DB, 1.02 P) when compared with the measurement of the maxillary second molar (0.54 mm MB, 0.43 mm DB, 0.62 mm P) are higher. We observed a similar tendency between the first (0.95 mm) and second (0.78 mm) mandibular molars. Kuttler^1^ as well as Stein and Corcoran^31^ investigated the distance between these two morphological entities, although without giving a tooth type specification. Kuttler reported a distance of 0.524 mm and 0.659 mm in individuals between 18–25 and 55 and more years of age,^1^ while Stein and Corcoran^31^ reported a distance of 0.724 mm, without making any distinction of tooth type; however, these results are similar to ours in the mandibular second molar.

Several authors describe different physiological foramina shapes such as kidney^32^ and asymmetrical, semilunar, hourglass or serrated.^33^ Because of their clinical relevance, in this investigation we classified the shapes into oval, round and irregular foramina. We were not able to define a specific physiological foramina shapein our study. However, predominantly, and of clinical relevance, the most commonly observed shape was oval, followed by round and irregular. The maxillary first molar physiological foramina oval shape results reported by Green^26^ (mesiobuccal 29%, distobuccal 39% and palatal 29%), Martos *et al.*^11^ (mesiobuccal 19.4%) and Abarca *et al.* (“maxillary molars” 50%),^24^ are lower when compared with the ones obtained in this investigation (mesiobuccal 69.2%, distobuccal 71.4% and palatal 70.4%). Only Arora and Tewari^34^ reported similar results (“minor apical foramen shape” 81%) to ours. Regarding the oval shape in the mandibular first molar (mesial 70.6%, distal 71.2%), other authors reported lower values in the mesial and distal roots. Green^26, 29^ reported an oval shape frequency of 35% (mesial) and 47% (distal), and of 43% (mesial) and 53% (distal) in two different studies. Martos *et al.*^11^ reported a 25.2% oval shape frequency in the mesial root. Similar results, when compared with ours, were obtained by Abarca *et al.*^24^ with 59% (“mandibular molars”) and Filpo-Perez *et al.*^12^ with 64.55% (“canal shape at the apical level of the distal canals of mandibular first molars presenting type I and II configurations of Vertucci”);^2^ yet, the measurements in these studies were made “1 mm before the apex”. To the best of our knowledge there is no evidence concerning the shape of the physiological foramina of maxillary and mandibular second molars. An oval shaped physiological foramen could play a decisive role in the outcome of an endodontic treatment. In case it could not be shaped into a round shape, the possibility of an incomplete shaping; thus, hermetic filing will diminish and the consequently accumulation of bacteria in the area would sustain the treatment failure. It is compulsory to take the entire root canal space into consideration when determining the final preparation size of the physiological foramina. Yet, a complete shaping/preparation of oval-shaped physiological foramina is clinically burdensome without significantly weakening or even perforating the root.^10^ Thus, the final physiological foramina preparation size recommendations (Table 5), based on the results obtained in the present study, should be considered not only according to the foramina morphological demands, but also according to their technically inherent limitations. Although, the statistically resulting standard deviations are equally distributed, the operator should be cautious when considering solely the mean wide and narrow diameters results of this investigation to determine the final preparation size of the physiological foramen.

## Conclusions

1. The mean distances between the physiological foramina and anatomical root apex of the roots were: 0.82 mm (MB), 0.81 mm (DB) and 1.02 mm (P) in maxillary first molars, 0.54 mm (MB), 0.43 mm (DB) and 0.63 mm (P) in maxillary second molars, 0.95 mm (M) and 1.05 mm (D) in mandibular first molars and 0.78 mm (M) and 0.81 mm (D) in mandibular second molars.
2. The mean narrow and wide physiological foramina diameters (respectively) were: 0.24/0.33 mm (MB), 0.22/0.31 mm (DB) and 0.33/0.42 mm (P) in maxillary first molars,0.24/0.41 mm (MB), 0.22/0.33 mm (DB) and 0.33/0.44 mm (P) in maxillary second molars, 0.24/0.39 mm (M) and 0.30/0.46 mm (D) in mandibular first molars and 0.25/0.47 mm (M) and 0.31/0.47 mm (D) mandibular second molars.
3. The physiological foramen shape frequencies were: 64.4%, 25.7% and 9.9% (MB); 67.8%, 18.1% and 14.1% (DB) and 70.9%, 20.1% and 9.0% (P) oval, round and irregular, respectively, in maxillary first molars,77.6%, 11.9% and 10.5% (MB); 68.4%, 23.1% and 8.5% (DB) and 73.1%, 20.2% and 6.7% (P) oval, round and irregular, respectively, in maxillary second molars,76.9%, 19.2% and 3.9% (M) and 86.8%, 11.8% and 1.4% (D) oval, round and irregular, respectively, in mandibular first molars and 82%, 14% and 4% (M) and 81.7%, 14.6% and 3.7% (D) oval, round and irregular, respectively, in mandibular second molars.

## Figures and Tables

**Table 1:** Teeth sample size and foramina number investigated

Teeth	Maxillary		Mandibular	
	*n*	Foramina	*n*	Foramina
First molar	179	612	118	439
Second molar	126	432	93	244
Total	305	1044	211	683

**Table 2:** Distance (mm) between the corresponding physiological foramina and anatomical root apex of maxillary and mandibular first and second molars

**1st MX**	MB (S)	MB (2)	MB (3)	DB (S)	DB (2)	P (S)	
Mean	0.82	0.84	0.93	0.81	0.8	1.02	
s.d.	0.28	0.30	0.36	0.26	0.26	0.33	
Max	1.63	1.74	2.3	1.49	1.49	2.10	
Min	0.27	0.17	0.23	0.22	0.22	0.42	
**2nd MX**	MB (S)	MB (2)	MB (3)	DB (S)	DB (2)	P (S)	P (2)
Mean	0.54	0.50	0.55	0.43	0.43	0.63	0.62
s.d.	0.28	0.10	0.22	0.21	0.16	0.32	0.10
Max	1.56	1.56	1.25	1.17	1.17	1.72	1.72
Min	0.19	0.15	0.14	0.08	0.08	0.09	0.09
**1st MA**	M (S)	M (2)	M (3–5)	D (S)	D (2)	D (3–4)	
Mean	0.95	0.97	0.95	1.05	1.01	1.06	
s.d.	0.29	0.25	0.22	0.24	0.41	0.27	
Max	1.98	1.98	1.76	1.91	2.63	2.52	
Min	0.54	0.57	0.54	0.59	0.56	0.59	
**2nd MA**	M (S)	M (2)	M (3–4)	D (S)	D (2)	D (3)	
Mean	0.78	0.73	0.70	0.81	0.60	0.81	
s.d.	0.37	0.32	0.32	0.34	0.23	0.34	
Max	1.86	1.86	2.13	1.71	0.96	1.71	
Min	0.24	0.24	0.35	0.28	0.19	0.28	

1st, first; 2, two foramina; 2nd, second; 3, three foramina; 3–5, three, four or five foramina; 3–4, three or four foramina; D, distal; DB, distobuccal; M, mesial; MA, mandibular; Max, maximum; MB, mesiobuccal; Min, minimum; MX, maxillary; P, palatal; S, single foramen; s.d., standard deviations.

**Table 3:** Statistical description of the physiological foramen diameter dimensions (mm) of root canals of first and second maxillary and mandibular molars in their respective root

1st MX	MB (S)		MB (2)		MB (3)		DB (S)		DB (2)		P (S)			
	N	W	N	W	N	W	N	W	N	W	N	W		
Mean	0.24	0.33	0.22	0.26	0.13	0.15	0.22	0.31	0.27	0.32	0.33	0.42		
s.d.	0.08	0.14	0.07	0.08	0.05	0.06	0.06	0.12	0.10	0.11	0.10	0.14		
Max	0.49	0.91	0.38	0.49	0.18	0.19	0.46	1.00	0.39	0.42	0.71	0.97		
Min	0.08	0.09	0.10	0.11	0.09	0.08	0.09	0.12	0.13	0.14	0.11	0.19		
*n*	109		134		9		177		4		179			
2nd MX	MB (S)		MB (2)		MB (3)		DB (S)		DB (2)		P (S)		P (2)	
	N	W	N	W	N	W	N	W	N	W	N	W	N	W
Mean	0.24	0.41	0.26	0.41	0.26	0.38	0.22	0.33	0.22	0.32	0.33	0.44	0.33	0.36
s.d.	0.07	0.21	0.09	0.08	0.08	0.15	0.07	0.14	0.17	0.11	0.11	0.15	0.03	0.16
Max	0.39	1.22	0.86	1.22	0.50	1.03	0.42	1.04	0.42	1.04	0.78	1.16	0.35	1.01
Min	0.09	0.11	0.09	0.11	0.09	0.16	0.03	0.09	0.03	0.09	0.16	0.21	0.30	0.35
*n*	67		106		9		117		6		119		8	
1st MA	M (S)		M (2)		M (3–5)		D (S)		D (2)		D (3–4)			
	N	W	N	W	N	W	N	W	N	W	N	W		
Mean	0.24	0.39	0.25	0.41	0.20	0.32	0.30	0.46	0.24	0.36	0.30	0.48		
s.d.	0.07	0.22	0.10	0.24	0.07	0.12	0.11	0.18	0.08	0.19	0.10	0.20		
Max	0.40	1.19	0.80	1.39	0.46	0.71	0.75	1.16	0.44	0.88	0.75	1.28		
Min	0.13	0.16	0.11	0.15	0.06	0.12	0.12	0.20	0.09	0.12	0.12	0.20		
*n*	26		146		114		76		128		51			
2nd MA	M (S)		M (2)		M (3–4)		D (S)		D (2)		D (3)			
	N	W	N	W	N	W	N	W	N	W	N	W		
Mean	0.25	0.47	0.25	0.45	0.20	0.33	0.31	0.47	0.27	0.38	0.31	0.47		
s.d.	0.09	0.23	0.10	0.24	0.06	0.16	0.09	0.16	0.06	0.10	0.09	0.16		
Max	0.44	1.12	0.60	1.13	0.39	0.39	0.57	0.92	0.40	0.49	0.57	0.92		
Min	0.08	0.19	0.02	0.02	0.08	0.08	0.11	0.20	0.19	0.20	0.11	0.20		
*n*	50		132		7		82		102		3			

1st, first; 2, two foramina; 2nd, second; 3, three foramina; 3–5, three, four or five foramina; 3–4, three or four foramina; D, distal; DB, distobuccal; M, mesial; MA, mandibular; Max, maximum; MB, mesiobuccal; Min, minimum; MX, maxillary; N, narrow diameter; *n*, total foramina per root; P, palatal; S, single foramen; s.d., standard deviations; W, wide diameter.

**Table 4:** Physiological foramen shape frequency (%) of first and second maxillary and mandibular molars

1st MX	M (S)	MB (2)	MB (3)	DB (S)	DB (2)	P (S)
Oval	64.4	69.4	44.4	67.8	75.0	70.9
Round	25.7	23.9	44.4	18.1	25.0	20.1
Irregular	9.9	6.7	11.2	14.1	0.0	9.0
*n*	109	134	9	177	4	179
2nd MX	M (S)	MB (2)	MB (3)	DB (S)	DB (2)	P (S)
Oval	77.6	75.5	66.7	68.4	100.0	73.1
Round	11.9	13.2	22.2	23.1	0.0	20.2
Irregular	10.5	11.3	11.1	8.5	0.0	6.7
*n*	67	106	9	117	6	119
1st MA	M (S)	M (2)	M (3–5)	D (S)	D (2)	D (3–4)
Oval	76.9	70.5	64.9	86.8	71.1	84.3
Round	19.2	21.9	29.8	11.8	24.2	9.8
Irregular	3.9	7.6	5.3	1.4	4.7	5.9
*n*	26	146	114	76	128	51
2nd MA	M (S)	M (2)	M (3–5)	D (S)	D (2)	D (3–4)
Oval	82.0	82.6	85.7	81.7	63.7	100.0
Round	14.0	10.6	14.2	14.6	27.5	0.0
Irregular	4.0	6.8	0.0	3.7	8.8	0.0
*n*	50	132	7	82	102	3

1st, first; 2, two foramina; 2nd, second; 3, three foramina; 3–5, three, four or five foramina; 3–4, three or four foramina; D, distal; DB, distobuccal; M, mesial; MA, mandibular; MB, mesiobuccal; MX, maxillary; *n*, total foramina per root; P, palatal; S; single foramen.

**Table 5:** Assumable recommendations for the final preparation size of the physiological foramen of the first and second maxillary and mandibular molars based on the results obtained

1st MX	Narrow diameter				Wide diameter			
	Mean	s.d.	IAF	%	Mean	s.d.	IAF	%
MB (S)	0.24	0.08	20	33.33	0.33	0.14	35	42.42
MB (2)	0.22	0.07	20	31.82	0.26	0.08	25	30.77
MB (3)	0.13	0.05	15	38.46	0.15	0.06	15	40.00
DB (S)	0.22	0.06	20	27.27	0.31	0.12	30	38.71
DB (2)	0.27	0.06	25	27.27	0.32	0.11	30	40.00
P (S)	0.33	0.10	35	30.30	0.42	0.14	40	33.33
2nd MX	Narrow diameter				Wide diameter			
	Mean	s.d.	IAF	%	Mean	s.d.	IAF	%
MB (S)	0.24	0.07	25	29.17	0.41	0.21	40	51.22
MB (2)	0.26	0.09	25	34.62	0.41	0.08	40	19.51
MB (3)	0.26	0.08	25	30.77	0.38	0.15	40	39.47
DB (S)	0.22	0.07	20	31.82	0.33	0.14	35	42.42
DB (2)	0.22	0.17	20	77.27	0.32	0.11	30	34.38
P (S)	0.33	0.11	35	33.33	0.44	0.15	45	34.09
1st MA	Narrow diameter				Wide diameter			
	Mean	s.d.	IAF	%	Mean	s.d.	IAF	%
M (S)	0.24	0.07	25	29.17	0.39	0.22	40	56.41
M (2)	0.25	0.10	25	40.00	0.41	0.24	40	58.54
M (3-5)	0.20	0.07	20	35.00	0.32	0.12	30	37.50
D (S)	0.30	0.11	30	36.67	0.46	0.18	45	39.13
D (2)	0.24	0.08	25	33.33	0.36	0.19	35	52.78
D (3-4)	0.30	0.10	30	33.33	0.48	0.20	50	41.67
2nd MA	Narrow diameter				Wide diameter			
	Mean	s.d.	IAF	%	Mean	s.d.	IAF	%
M (S)	0.25	0.09	25	36.00	0.47	0.23	45	48.94
M (2)	0.25	0.10	25	40.00	0.45	0.24	45	53.33
M (3-4)	0.20	0.06	20	30.00	0.33	0.16	35	48.48
D (S)	0.31	0.09	30	29.03	0.47	0.16	45	34.04
D (2)	0.27	0.06	25	22.22	0.38	0.10	40	26.32
D (3)	0.31	0.09	30	29.03	0.47	0.16	45	34.04

The determined size of the IAF was calculated according to the mean values of the narrow and wide diameters. Friction at the tip of an instrument at the physiological foramen would be clinically the only conceivable advise that an operator could have to determine the diameter size of the physiological foramen or IAF size. However, due to the limitations inherent to this procedure, it is only the size of the narrow diameter that can be clinically detected; yet, the final preparation size of the physiological foramen should be determined according to the dimension of the wide, and not of the narrow, diameter. The mean figures (%) indicate the possible IAF calculation error margin, according to the resulting s.d., when employing the given IAF recommendations based on the dimensions obtained of either the narrow or wide diameters. 1st, first; 2, two foramina; 2nd, second; 3, three foramina; 3–5, three, four or five foramina; 3–4, three or four foramina; IAF, initial apical files; MA, mandibular; MX, maxillary; S, single foramen; s.d., standard deviations.
